# Mesenchymal Stem Cell-Derived Microvesicles Support Ex Vivo Expansion of Cord Blood-Derived CD34^+^ Cells

**DOI:** 10.1155/2016/6493241

**Published:** 2016-03-06

**Authors:** Hui Xie, Li Sun, Liming Zhang, Teng Liu, Li Chen, Aiqi Zhao, Qian Lei, Fei Gao, Ping Zou, Qiubai Li, An-yuan Guo, Zhichao Chen, Hongxiang Wang

**Affiliations:** ^1^Institute of Hematology, Union Hospital, Tongji Medical College, Huazhong University of Science and Technology, Wuhan 430022, China; ^2^Department of Hematology, The Second Hospital of Hebei Medical University, Shijiazhuang 050000, China; ^3^Department of Hematology, The Central Hospital of Jingzhou, Jingzhou 434020, China; ^4^Department of Biomedical Engineering, Key Laboratory of Molecular Biophysics of the Ministry of Education, College of Life Science and Technology, Huazhong University of Science and Technology, Wuhan 430074, China; ^5^Department of Hematology, The Central Hospital of Wuhan, Wuhan 430012, China

## Abstract

Mesenchymal stem cells (MSCs) are known to support the characteristic properties of hematopoietic stem and progenitor cells (HSPCs) in the bone marrow hematopoietic microenvironment. MSCs are used in coculture systems as a feeder layer for the ex vivo expansion of umbilical cord blood (CB) to increase the relatively low number of HSPCs in CB. Findings increasingly suggest that MSC-derived microvesicles (MSC-MVs) play an important role in the biological functions of their parent cells. We speculate that MSC-MVs may recapitulate the hematopoiesis-supporting effects of their parent cells. In the current study, we found MSC-MVs containing microRNAs that are involved in the regulation of hematopoiesis. We also demonstrated that MSC-MVs could improve the expansion of CB-derived mononuclear cells and CD34^+^ cells and generate a greater number of primitive progenitor cells in vitro. Additionally, when MSC-MVs were added to the CB-MSC coculture system, they could improve the hematopoiesis-supporting effects of MSCs. These findings highlight the role of MSC-MVs in the ex vivo expansion of CB, which may offer a promising therapeutic approach in CB transplantation.

## 1. Introduction

Hematopoietic stem cell transplantation (HSCT) has become a common procedure in the treatment of malignant hematologic diseases [1]. Compared with bone marrow or mobilized peripheral blood progenitor cells from adult donors, umbilical cord blood (CB) has emerged as an attractive source of hematopoietic stem and progenitor cells (HSPCs) for HSCT. They have several advantages, such as easy acquisition, ready availability, and reduced incidence and severity of graft versus host disease as well as less stringent requirements for human leukocyte antigen matches between donor and recipient [2]. However, a major limitation in CB transplantation is the insufficient number of total nucleated cells (TNCs) and CD34^+^ cells available for transplantation [3]. This is thought to be the main reason for the delayed neutrophil and platelet engraftment and the high risk of engraftment failure, which are often associated with CB transplantation [4].

To overcome this limitation, substantial effort has been dedicated to developing strategies to increase the number of HSPCs in CB prior to infusion. Mesenchymal stem cells (MSCs) are adult stem cells of mesodermal origin that have been identified as one major component of the bone marrow hematopoietic microenvironment [5]. It has been demonstrated that MSCs can secrete or express a broad range of hematopoiesis-regulating molecules that can regulate characteristic functional properties of HSPCs [6]. In addition, researchers used MSCs as a feeder layer for the ex vivo expansion of CB cells [7]. By using this coculture system, markedly improved expansion efficiency and better maintenance of cell “stemness” have been achieved compared with those with a liquid culture system supplemented with a combination of growth factors [8].

Accumulating evidence suggests that the therapeutic effects of MSCs are mainly attributable to their paracrine effects [9, 10]. It is now recognized that, apart from soluble factors, MSCs can also secrete a large number of microvesicles (MVs). MVs are important mediators of cell-to-cell communication that have long been underappreciated [11]. They are heterogeneous mixtures of vesicular, organelle-like structures that are released by various cell types. They mainly include exosomes derived from the endosomal compartment and microparticles (also called ectosomes) derived directly from budding of the cell plasma membrane [12, 13]. Proteins, lipids, messenger RNAs (mRNAs), and microRNAs (miRNAs) derived from their parent cells are selectively packaged into MVs and can be transferred between cells via MVs. By the horizontal transfer of their bioactive cargo, MVs may mediate reprogramming of the target cells [14, 15]. Rapidly accumulating evidence has suggested that MVs play important roles in a broad range of physiological and pathological processes [16]. Ratajczak et al. discovered that MVs derived from embryonic stem cells significantly improved the ex vivo expansion of hematopoietic progenitor cells (HPCs) and upregulated the expression of early pluripotent and early hematopoietic stem cell (HSC) markers in HPCs, suggesting that MVs may be an important regulator of the characteristic functional properties of HSPCs [14]. Moreover, data from both in vitro and in vivo experiments have suggested that MSC-derived MVs (MSC-MVs) are potential key mediators of the biological function of MSCs [17]. The therapeutic effects of MSC-MVs have been confirmed in several animal models of tissue injuries [18–21]. In these studies, the therapeutic effects of MSC-MVs were found to be comparable to those of their parent cells. Thus, we speculate that MSC-MVs may also mimic the beneficial effects of MSCs in the ex vivo expansion of CB. In the current study, we tested MSC-MVs for their potential to improve the ex vivo expansion of CB.

## 2. Materials and Methods

### 2.1. Cell Culture

#### 2.1.1. Primary Culture of Human Bone Marrow-Derived MSCs

This study was approved by the Ethical Committee of Tongji Medical College, Huazhong University of Science and Technology. Human bone marrow aspirates were collected from healthy donors with informed consent. Low-density mononuclear cells (MNCs) were separated by Ficoll gradient centrifugation (Haoyang Biological, Tianjin, China) and cultured in Dulbecco's modified Eagle's medium (DMEM; Hyclone, Waltham, MA, USA) supplemented with 10% fetal bovine serum (FBS; Gibco, Carlsbad, CA, USA) and 1% penicillin–streptomycin (Beyotime, Shanghai, China) in a humidified incubator under an atmosphere of 5% CO_2_/95% air at 37°C. Nonadherent cells were removed by replacing the medium after 48 h of incubation. Cell passaging was performed when the monolayer of adherent cells reached 80% confluence with 0.25% trypsin-EDTA (Beyotime).

#### 2.1.2. Culture of Human Umbilical Vein Endothelial Cells

Human umbilical vein endothelial cells (HUVECs) were purchased from the American Type Culture Collection (ATCC, Rockville, MD, USA). The cells were cultured in DMEM supplemented with 10% FBS and 1% penicillin-streptomycin antibiotic in a humidified incubator with an atmosphere of 5% CO_2_/95% air at 37°C. Cell passaging was performed when the monolayer of adherent cells reached 90% confluence.

### 2.2. Isolation and Characterization of MSC-MVs

MSC-MVs were harvested as previously described with some modifications [33]. Briefly, human bone marrow-derived MSCs from the third to fifth passages were used for MV isolation. Before MV isolation, the cell culture medium was replaced by serum-free DMEM for an additional 24 h of incubation. The conditioned medium was collected and centrifuged at 1500 ×g for 20 min to remove cell debris. MVs were pelleted by centrifugation of the supernatant at 16,000 ×g for 1 h at 4°C. The supernatant was removed, and then the pelleted MVs were washed with PBS and pelleted again by centrifugation at 16,000 ×g for 1 h at 4°C. Finally, the supernatant was removed, and the pelleted MVs were resuspended with PBS and stored at −80°C for further experiments. The quantity of MVs was determined by measuring the total protein content of MVs using a BCA protein assay kit (Beyotime) following the manufacturer's instructions. MVs were identified by transmission electron microscopic and flow cytometric analyses.

For transmission electron microscopic analysis, pelleted MVs were fixed in 3% glutaraldehyde for 2 h, and then in 1% osmium tetroxide. Following serial dehydration, MVs were immersed in propylene oxide and then embedded in resin. Sections of 0.1 *μ*m thickness were prepared from the MV pellets. Microscopic photographs (×25,600) of MVs were taken with a Fei.Tecnai.GZ.12 transmission electron microscope (FEI, Hillsboro, OR, USA). Flow cytometric analysis was performed as described below.

### 2.3. Microarray and Bioinformatic Analyses of miRNAs in MSC-MVs

Total RNA was isolated from MSC-MVs using a mirVana miRNA isolation kit (Ambion, Austin, TX, USA), in accordance with the manufacturer's instructions. RNA concentration and RNA integrity were analyzed on an Agilent Bioanalyzer 2100 (Agilent Technologies, Santa Clara, CA, USA). With a miRNA Complete Labeling and Hyb Kit (Agilent Technologies), miRNAs were fluorescently labeled and then hybridized in a hybridization oven (Agilent Technologies). Slides were scanned with an Agilent Microarray Scanner (Agilent Technologies) with the Feature Extraction software 10.7 (Agilent Technologies). Raw data were exported to the GeneSpring GX11.0 software (Agilent Technologies) for quartile normalization and further analyses.

The expression levels of miRNAs were calculated as the base 2 logarithm of the nominalized signals and miRNAs with expression >6 were considered to be expressed at a relatively high level. The potential targets of expressed miRNAs were predicted using miRDB (Version 6.2), using a target prediction score ≥90. To investigate the roles of miRNAs in biological processes, all of the predicted targets were analyzed by Gene Ontology enrichment analysis using DAVID 6.7. Genes that inhibit the Wnt/*β*-catenin pathway were obtained from KEGG (map04310). Genes expressed in CB-derived HSCs were obtained from a gene expression profile in the NCBI GEO database (GSE58299). We used a bioconductor to screen out candidate genes that are known to inhibit the Wnt/*β*-catenin pathway and are expressed in CB-derived HSCs. Then, miRNAs that may target these candidate genes were predicted using miRDB.

### 2.4. Collection and Purification of CB-Derived MNCs and CD34^+^ Cells

Human CB samples were collected from normal full-term pregnancies after obtaining informed consent. The samples were processed within 4 h postpartum. Blood was mixed with hydroxyethyl starch (Sigma) to remove the majority of red blood cells. Low-density MNCs were isolated by Ficoll gradient centrifugation (Haoyang Biological). CD34^+^ cells were enriched from MNCs using bead-conjugated anti-CD34 antibody (Miltenyi Biotec Inc., Auburn, CA, USA) with the Magnetic-Activated Cell Sorting system (Miltenyi Biotec Inc.), in accordance with the manufacturer's instructions. The average percentage of CD34^+^ cells among the CD34^+^-selected cells was higher than 90%, as determined by flow cytometry.

### 2.5. Ex Vivo Expansion of CB-Derived MNCs and CD34^+^ Cells

CB-MNCs were plated under various culture conditions at 5 × 10^5^ cells/mL for 28 days. The culture conditions were classified into four groups: (1) The control group: Roswell Park Memorial Institute (RPMI) 1640 medium (Hyclone) supplemented with 10% FBS (Gibco) and recombinant cytokines consisting of 100 ng/mL each of stem cell factor (SCF; Peprotech, Rocky Hill, NJ, USA), thrombopoietin (TPO; Peprotech), Flt3-ligand (Flt3-L; Peprotech), and granulocyte colony-stimulating factor (G-CSF; Peprotech). (2) The MV group: RPMI 1640 medium supplemented with 10% FBS, recombinant cytokines (the same concentrations as above), and MSC-MVs (10 *μ*g/mL) without MSCs as a cell feeder layer. (3) The MSC group: RPMI 1640 medium supplemented with 10% FBS, recombinant cytokines (the same concentrations as above), and MSCs as a cell feeder layer. (4) The MSC+MV group: RPMI 1640 medium supplemented with 10% FBS, recombinant cytokines (the same concentrations as above), MSC-MVs (10 *μ*g/mL), and MSCs as a cell feeder layer. Twice a week, half the volume of fresh medium and cytokines with or without MVs was exchanged in all groups. On days 7, 14, 21, and 28 of expansion, the cells were carefully counted to determine the total cell number. Meanwhile, on day 7 of expansion, the cells were characterized by the expression of the cell surface markers CD45, CD3, CD19, CD14, CD15, CD56, CD34, and CD38 by flow cytometry.

CD34^+^-selected cells were plated under various culture conditions at 2 × 10^4^ cells/mL for 14 days. The culture conditions were classified into four groups as described above. Twice a week, half the volume of fresh medium and cytokines with or without MVs was exchanged in all groups. On days 3, 7, 10, and 14 of expansion, the cells were carefully counted to determine the total cell number and the proportion of CD34^+^ cells was analyzed by flow cytometry. In addition, at different time points, cells were subjected to the colony-forming cell (CFC) assay, cobblestone-area-forming cell (CAFC) assay, endothelial adherence assay, transwell migration assay, and western blot analysis, as described below.

### 2.6. Flow Cytometric Analyses

Flow cytometric analyses were performed to (1) determine the size distribution and phenotype of MSC-MVs; (2) determine the phenotype of expanded CB-MNCs; and (3) determine the proportion of CD34^+^ cells among expanded CD34^+^-selected cells. All of the antibodies used for flow cytometric analyses were purchased from BioLegend (San Diego, CA, USA).

MSC-MVs were assayed for their size distribution and expression of CD73, CD105, CD29, CD44, and CD90 as previously described [33]. Expanded CB-MNCs were analyzed using phycoerythrin- (PE-) or peridinin chlorophyll protein- (PerCP-) conjugated anti-CD45, -CD3, -CD19, -CD14, -CD15, -CD56, -CD34, and -CD38 antibodies. Expanded CD34^+^-selected cells were analyzed using PE-conjugated anti-CD34 antibody. Measurements were performed with a FACS Aria II cytometer (BD Bioscience, San Jose, CA, USA) and analyzed with FlowJo software, version 7.6 (TreeStar Inc., Ashland, OR, USA). The proportions of CD45^+^CD3^+^, CD45^+^CD19^+^, CD45^+^CD14^+^, CD45^+^CD15^+^, CD45^+^CD56^+^, and CD34^+^CD38^−^ cells are expressed as percentages of TNCs. The number of CD34^+^ cells was calculated according to the mean number of TNCs.

### 2.7. CFC Assay

For various culture conditions assayed, triplicate CFC assays were performed. Briefly, cells were harvested after 7 and 14 days of expansion and then cultured in MethoCult GF H4435 methylcellulose medium (StemCell Technologies, Vancouver, BC, Canada), following the manufacturer's instructions. CFC assays were cultured for 14 days in an atmosphere of 5% CO_2_/95% air at 37°C. The cells that formed clones (containing more than 50 cells) were counted under an inverted microscope (Olympus, Tokyo, Japan) and identified as CFCs. CFC frequencies and total CFC numbers per group were determined. Cytospins of each culture were prepared and subjected to Wright-Giemsa staining and then observed using a light microscope (Olympus).

### 2.8. CAFC Assay

For various culture conditions assayed, triplicate CAFC assays were performed. The frequencies of CAFCs in samples were determined by limiting dilution analysis, as described previously with some modifications [34]. Briefly, MSCs were seeded at 1000 cells/well in a 96-well plate 1 week before the assay. CAFC medium was prepared as follows: Iscove's Modified Dulbecco's Medium (Hyclone) supplemented with 20% horse serum (Sigma), 10 ng/mL interleukin-3 (Sigma), 10 ng/mL G-CSF (Peprotech), 10^−5 ^M hydrocortisone (Sigma), 10^−5^ M 2-mercaptoethanol (Sigma), and 100 U/mL penicillin-streptomycin (Beyotime). The cells were harvested after 7 and 14 days of expansion and then inoculated onto MSCs at various dilutions. We used six threefold dilutions per sample. The starting cell concentration was 60,000 cells/well, and the concentration was gradually decreased to 25 cells/well. For each dilution, 20 replicated wells were tested. Half of the volume of fresh CAFC medium was exchanged twice a week. Hematopoietic clones (containing more than six cells) under the stromal layer were scored under an inverted microscope (Olympus) after 5 weeks of incubation. The CAFC frequency was calculated using Poisson statistics [34] as described previously and the total number of CAFCs per group was determined.

### 2.9. Transwell Migration Assay

The transwell migration assay was performed to evaluate the chemotactic responses of the ex vivo-expanded CD34^+^ cells to stromal cell-derived factor-1 (SDF-1). The bottom compartment of the chamber (Corning Costar, New York, NY, USA) was filled with serum-free PRMI1640 medium supplemented with 100 ng/mL SDF-1 (Peprotech). The upper compartment was seeded with cells collected after 7 days of ex vivo expansion (1 × 10^5^/well). Triplicate wells were set up for each group. For each experimental set, some wells were filled with RPMI1640 medium without SDF-1 to assess spontaneous migration. Some wells were filled with RPMI1640 medium supplemented with 10% FBS and served as positive control. After 4 h of incubation, the upper compartment was removed and the cells in the lower compartment were harvested and counted. The migration rate of each group was calculated using the following formula: (number of migrated cells − number of spontaneously migrated cells)/total input number × 100%.

### 2.10. Endothelial Adherence Assay

HUVECs were seeded at 3 × 10^3^ cells/well in a 96-well plate 24 h before the assay. For various culture conditions, after 7 days of ex vivo expansion, cells were harvested and plated on HUVECs at 5 × 10^5^ cells/well. Five replicated wells were set up for each group. Cells plated on HUVECs in the presence of SDF-1 (100 ng/mL) served as positive control. After 4 h of incubation, nonadherent cells were removed from the plate by gentle washing with PBS and the number of adherent cells was determined by 3-(4,5-dimethylthiazol-2-yl)-2,5-diphenyltetrazolium bromide (MTT) assay. Briefly, 5 mg/mL MTT (Sigma) was added and the culture was maintained for another 4 h. The formazan was dissolved in dimethyl sulfoxide and the optical density (OD) at 490 nm was measured using a microplate reader (Thermo Scientific, Waltham, MA, USA).

### 2.11. Western Blot Analysis

For various culture conditions, after 7 days of ex vivo expansion, cells were collected and solubilized in sodium dodecyl sulfate lysis buffer. Protein samples (40 *μ*g) were electrophoresed and then transferred to polyvinylidene fluoride membranes. Immunoblotting was performed by incubating membranes with anti-*β*-catenin (dilution: 1 : 400; Sigma) and anti-*β*-actin (dilution: 1 : 400; Sigma) primary antibodies overnight at 4°C. After being washed in PBS, membranes were incubated for 1 h with a secondary peroxidase-conjugated antibody (dilution: 1 : 1000; Santa Cruz Biotechnology, Santa Cruz, CA, USA) at room temperature. Enhanced chemiluminescence was performed in accordance with the manufacturer's instructions (Beyotime).

### 2.12. Statistical Analysis

All collected data are presented as the mean ± standard deviation (SD) and were analyzed using one-way analysis of variance. *P* < 0.05 was considered significant.

## 3. Results

### 3.1. Characterization of MSC-MVs

**Figure 1:**
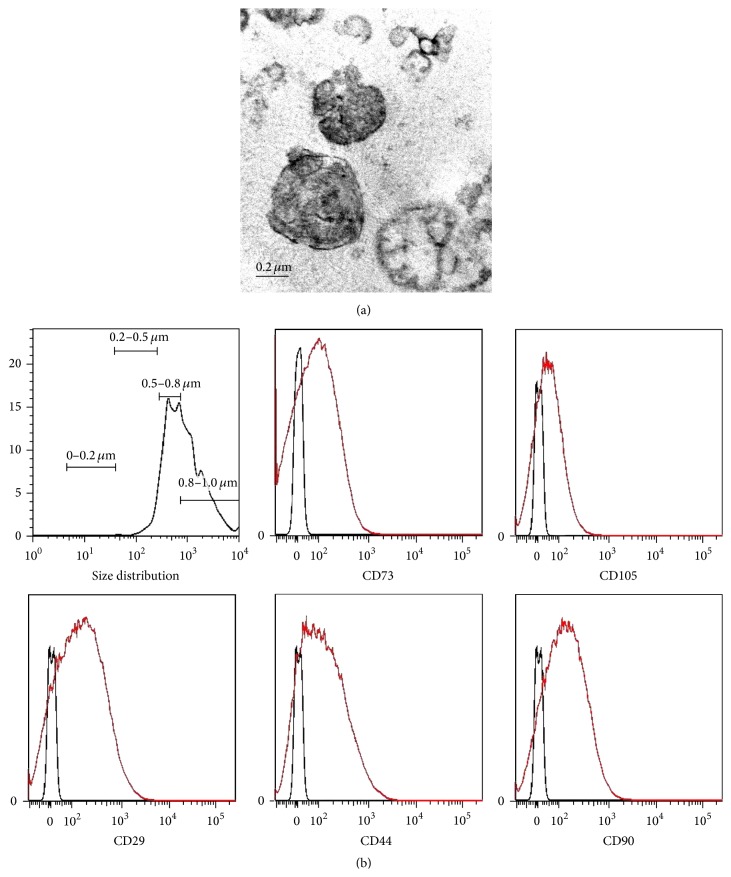
Characterization of MSC-MVs. (a) Typical morphology of MSC-MVs observed under a transmission electron microscope. Scale bar = 0.2 *μ*m. (b) Representative flow cytometric histograms of MV surface marker expression.

MSC-MVs were observed under a transmission electron microscope. They exhibited a spheroid shape with a diameter between 100 and 1000 nm (Figure 1(a)). In the flow cytometric analysis of MSC-MVs, calcein-AM was used to determine the integrity of MVs and to avoid the contamination staining of debris, as described previously [33]. As shown in Figure 1(b), our MSC-MV sample collected following the differential centrifugation protocol consisted of vesicles with different size distributions. MSC-MVs exhibited surface marker expression profiles similar to those of their parent cells; specifically, they were positive for CD73, CD105, CD29, CD44, and CD90.

### 3.2. Bioinformatic Analyses of miRNAs in MSC-MVs

**Figure 2:**
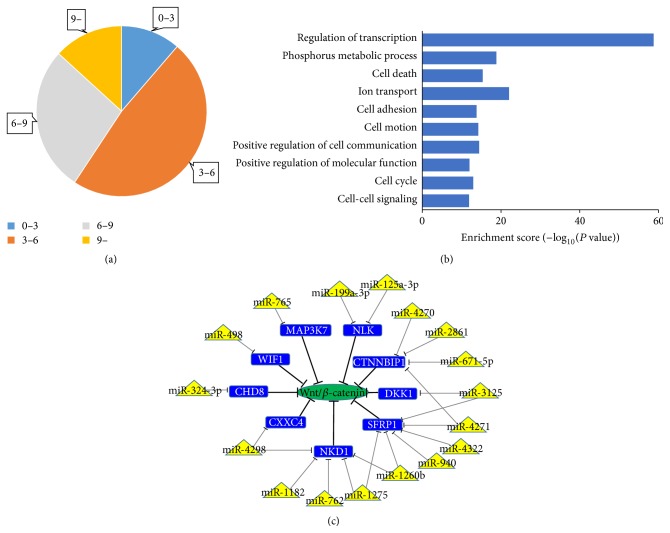
Bioinformatic analyses of miRNAs in MSC-MVs. (a) The overall expression level of miRNAs in MSC-MVs. (b) Gene Ontology enrichment analysis of the potential targets of expressed miRNAs in MSC-MVs. (c) A schematic illustration representing miRNAs and potential targets involved in regulation of the Wnt/*β*-catenin pathway. Blue rectangles: genes that are reported to inhibit the Wnt/*β*-catenin pathway and are expressed in CB-derived HSCs. Yellow triangles: relatively highly expressed miRNAs in MSC-MVs. Green ellipses: Wnt/*β*-catenin pathway. Black and gray lines indicate inhibitory effects.

Using microarray analysis, we detected 469 known human miRNAs expressed in MSC-MVs (Table S1, Supporting Information, in supplementary materials available online at http://dx.doi.org/10.1155/2016/6493241). The overall expression level of miRNAs is shown in Figure 2(a); miRNAs with expression >6 were considered expressed at a relatively high level. The potential targets of the expressed miRNAs were analyzed by Gene Ontology enrichment analysis. The results showed that MSC-MVs contained miRNAs that are involved in various biological processes, some of which are presented in Figure 2(b). By reviewing the literature, we found that MSC-MVs contain some miRNAs that are reported to be involved in the regulation of hematopoiesis (Table 1) [22–32]. It has been reported that the canonical Wnt/*β*-catenin signaling pathway is crucial for the regulation of HSC functions. Thus, we investigated whether MSC-MVs contain miRNAs that may target the Wnt/*β*-catenin pathway. We found nine candidate genes that are reported to inhibit this pathway and are expressed in CB-derived HSCs. A total of 811 miRNAs that may target these candidate genes were predicted using miRDB. Among these, we found that 17 were expressed at a relatively high level in MSC-MVs (Figure 2(c)).

### 3.3. The Effects of MSC-MVs on Ex Vivo Expansion of CB-MNCs

**Figure 3:**
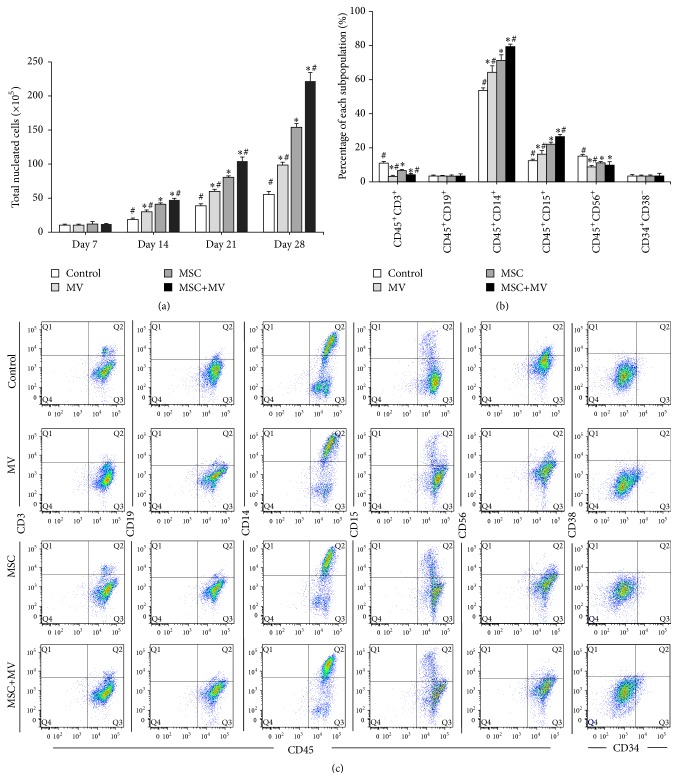
Ex vivo expansion of CB-MNCs. CB-MNCs were maintained under four different culture conditions: control, MV, MSC, and MSC+MV. (a) Growth profile of TNCs (*n* = 3), ^*∗*^ *P* < 0.05 compared with the control group, ^#^ *P* < 0.05 compared with the MSC group. (b) Immunophenotype of subpopulations of expanded CB-MNCs analyzed by flow cytometry on day 7 (*n* = 3), ^*∗*^ *P* < 0.05 compared with the control group, ^#^ *P* < 0.05 compared with the MSC group. (c) Representative flow cytometric dot blot diagrams of each subpopulation.

We first investigated the effects of MSC-MVs on the ex vivo expansion of unselected CB cells. The optimal dosage of MSC-MVs was determined at 10 *μ*g/mL based on an in vitro screen for the expansion of CB-MNCs with different concentrations of MSC-MVs (1, 5, 10, and 100 *μ*g/mL) (Figure S1, Supporting Information). CB-MNCs were separated into four groups: control, MV, MSC, and MSC+MV, as described above. As shown in Figure 3(a), no differences were found in the number of TNCs in each group on day 7. However, MSC-MVs clearly increased the number of TNCs compared with that in the control group on days 14, 21, and 28. Although the promoting effect of MSC-MVs was not as potent as that of their parent cells, the proliferation rate of TNCs was substantially greater when both MVs and MSCs were present (Figure 3(a)). The numbers of TNCs after 28 days of ex vivo expansion were 55.3 ± 4.5 × 10^5^ for the control group, 98.7 ± 4.0 × 10^5^ for the MV group, 154.0 ± 5.6 × 10^5^ for the MSC group, and 221.7 ± 12.6 × 10^5^ for the MSC+MV group.

Flow cytometric analysis was performed to evaluate the effects of MSC-MVs on the phenotypes of the expanded cells on day 7. The results showed that the percentages of CD45^+^CD14^+^ (monocytes) and CD45^+^CD15^+^ (granulocytes) cells in the MSC-MV group were the highest, followed by the MSC group, the MV group, and the control group, indicating that MSC-MVs preferentially promoted the expansion of monocytes and granulocytes (Figure 3(b)). In addition, cells cultured in the control group included more CD45^+^CD3^+^ (T lymphocytes) and CD45^+^CD56^+^ (natural killer cells) cells than the other groups, indicating that both MSCs and MVs were able to inhibit the proliferation of T lymphocytes and NK cells (Figure 3(b)). Interestingly, the effects inhibiting the proliferation of T lymphocytes and NK cells were stronger in the MV group than in the MSC group (Figure 3(b)). However, there were no significant differences in the percentages of CD34^+^CD38^−^ cells among the four groups (Figure 3(b)).

### 3.4. The Effects of MSC-MVs on Ex Vivo Expansion of CD34^+^-Selected CB Cells

**Figure 4:**
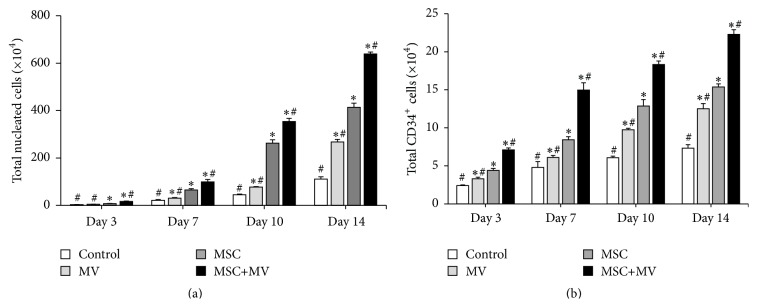
Ex vivo expansion of CD34^+^-selected cells. CD34^+^-selected cells were maintained under four different culture conditions: control, MV, MSC, and MSC+MV. (a) Growth profile of TNCs (*n* = 3), ^*∗*^ *P* < 0.05 compared with the control group, ^#^ *P* < 0.05 compared with the MSC group. (b) Growth profile of CD34^+^ cells (*n* = 3), ^*∗*^ *P* < 0.05 compared with the control group, ^#^ *P* < 0.05 compared with the MSC group.

As we demonstrated that MSC-MVs promoted the expansion of unselected CB cells, we further investigated the effects of MSC-MVs on the expansion of CD34^+^-selected cells. CD34^+^-selected cells were isolated from CB and separated into four groups: control, MV, MSC, and MSC+MV. The results showed that the rate of proliferation of TNCs in the MSC+MV group was the highest, followed by those in the MSC group, the MV group, and the control group, throughout the culture period (Figure 4(a)). The numbers of TNCs after 14 days of expansion were 111.0 ± 9.5 × 10^4^ for the control group, 267.7 ± 10.6 × 10^4^ for the MV group, 414.0 ± 17.1 × 10^4^ for the MSC group, and 639.0 ± 7.9 × 10^4^ for the MSC+MV group. Next, we investigated cell surface markers of HSPCs among expanded cells by staining the cells with CD34. As shown in Figure 4(b), at four time points, more CD34^+^ cells were harvested in the MV group than in the control group. In addition, the proliferation rate of CD34^+^ cells in the MSC coculture system was significantly enhanced by the addition of MSC-MVs. The total numbers of CD34^+^ cells after 14 days of expansion were 7.3 ± 0.5 × 10^4^ for the control group, 12.5 ± 0.7 × 10^4^ for the MV group, 15.4 ± 0.4 × 10^4^ for the MSC group, and 22.3 ± 0.6 × 10^4^ for the MSC+MV group.

### 3.5. The Effects of MSC-MVs on the Generation of CFCs and CAFCs and *β*-Catenin Expression in Expanded CD34^+^ Cells

**Figure 5:**
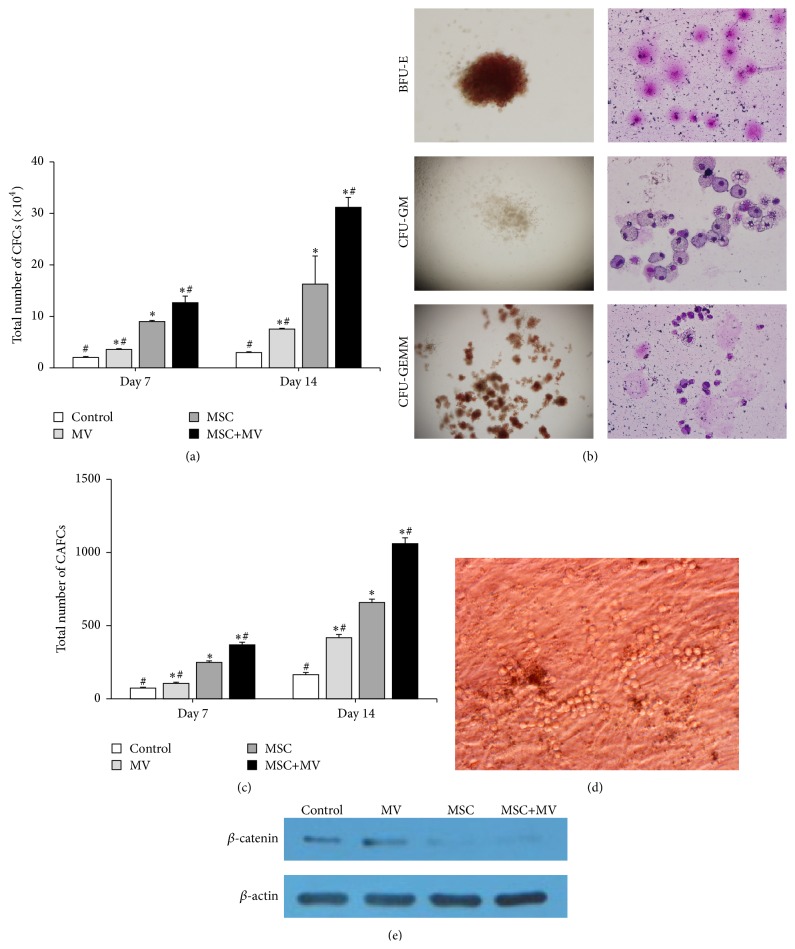
The effects of MSC-MVs on the generation of CFCs and CAFCs and the expression of *β*-catenin in the expanded CD34^+^ cells. (a) Total number of CFCs generated in the four groups after 7 and 14 days of expansion (*n* = 3), ^*∗*^ *P* < 0.05 compared with the control group, ^#^ *P* < 0.05 compared with the MSC group. (b) Typical morphology of BFU-E, CFU-GM, and CFU-GEMM, observed using an inverted microscope and after Wright-Giemsa staining. (c) Total number of CAFCs generated in the four groups after 7 and 14 days of expansion (*n* = 3), ^*∗*^ *P* < 0.05 compared with the control group, ^#^ *P* < 0.05 compared with the MSC group. (d) Representative images of CAFCs. (e) Representative gel photograph of *β*-catenin protein expression.

The CFC assay was performed to measure the frequency of progenitor cells that were able to produce a large number of progenies [34]. After 7 and 14 days of expansion, the progenies of CD34^+^ cells under various culture conditions were harvested and seeded in semisolid methylcellulose, as described above. The results showed that, after 7 and 14 days of expansion, the progeny of CD34^+^ cells in the MSC+MV group generated the highest number of CFCs (12.7 ± 1.3 × 10^4^ and 31.2 ± 1.9 × 10^4^), followed by those in the MSC group (9.0 ± 0.2 × 10^4^ and 16.3 ± 5.5 × 10^4^), the MV group (3.6 ± 0.2 × 10^4^ and 7.5 ± 0.2 × 10^4^), and the control group (2.0 ± 0.2 × 10^4^ and 3.0 ± 0.2 × 10^4^) (Figure 5(a)). In addition, morphological analysis showed that the expanded cells in all four groups possessed the ability to produce clonogenic progenitor cells: burst-forming unit erythroid (BFU-E), colony-forming unit granulocyte/macrophage (CFU-GM), and colony-forming unit granulocyte/erythroid/macrophage/megakaryocyte (CFU-GEMM) (Figure 5(b)).

The CAFC assay is commonly used to measure the frequency of early hematopoietic precursor cells in vitro [34]. CAFCs found after 5 weeks of culture are considered to represent relatively primitive HPCs [35]. As shown in Figures 5(c) and 5(d), after 7 and 14 days of expansion, the progeny of CD34^+^ cells in the MSC+MV group generated the highest number of CAFCs (369 ± 18 and 1061 ± 39), followed by those in the MSC group (248 ± 11 and 658 ± 22), the MV group (105 ± 9 and 418 ± 22), and the control group (73 ± 7 and 165 ± 15). These data suggest that MSC-MVs could promote the generation of CFCs and CAFCs.

It has been reported that the canonical Wnt/*β*-catenin signaling pathway is crucial for the regulation of HSPC functions [36], so we tested the expression of *β*-catenin in the expanded CD34^+^ cells. Compared with the control group, the MSC group showed decreased expression of *β*-catenin, while the MV group showed increased expression of it. Furthermore, the addition of MSC-MVs to the MSC coculture system seemed to increase the expression of *β*-catenin compared with that in the MSC group (Figure 5(e)).

### 3.6. The Effects of MSC-MVs on Endothelial Adherence and Migration of Expanded CD34^+^ Cells

**Figure 6:**
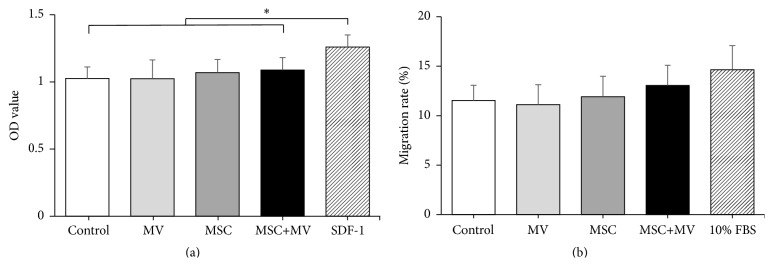
The effects of MSC-MVs on the endothelial adherence and migration of the expanded CD34^+^ cells. (a) The expanded cells were seeded on a monolayer of HUVECs, and the number of adherent cells was determined by MTT assay. Five replicated wells were set up for each group, SDF-1 served as positive control, ^*∗*^ *P* < 0.05. (b) The migration abilities of the expanded cells were assessed by a transwell assay. Three replicated wells were set up for each group; 10% FBS served as positive control.

The adherence of HSCs to bone marrow endothelium under the shear flow of blood and the subsequent transendothelial migration toward bone marrow extravascular space are key processes in the initial phase of the engraftment of HSCs after transplantation [37]. Therefore, we further studied the effects of MSC-MVs on the endothelial adherence and migration of the expanded CD34^+^ cells. The results showed no significant differences among the four groups in their adherence to HUVECs and chemotactic responses to SDF-1 (Figures 6(a) and 6(b)).

## 4. Discussion

It is now recognized that MSCs can secrete a large number of MVs, which play an important role in the biological functions of MSCs [17]. Assuming that MSC-MVs may mimic the hematopoiesis-supporting effects of MSCs, we investigated whether MSC-MVs could improve the ex vivo expansion of CB. In the current study, we first isolated MSC-MVs from the conditioned medium of MSCs. In line with current practices, microparticles (100–1000 nm in diameter) can be pelleted in the range of 10,000–20,000 ×g, which is in accordance with our centrifugation protocol, while smaller exosomes (30–100 nm in diameter) can only be pelleted in the range of 100,000–120,000 ×g [38]. Additionally, transmission electron microscopic and flow cytometric analyses showed that the diameters of our MSC-MV sample ranged from 100 to 1000 nm, suggesting that what we isolated from the conditioned medium of MSCs mainly included microparticles.

To the best of our knowledge, this is the first time that the possibility of using MSC-MVs as a new tool or additive for the ex vivo expansion of CB has been studied. Our results show that MSC-MVs could promote the expansion of both unselected and CD34^+^-selected CB cells in vitro and generate greater numbers of primitive cells compared with that in the control group. These results suggest that MSC-MVs may be one of the cues provided in vivo by MSCs in the hematopoietic microenvironment that help to maintain the characteristic functional properties of HSPCs. It is noteworthy that the promoting effects of MSC-MVs were not as potent as those of their parent cells, which is in line with our expectations because, apart from MVs, MSCs would also release a combination of trophic soluble factors that are able to support HSPC functions. However, the rate of expansion of CD34^+^-selected CB cells and the number of generated primitive cells were substantially higher when MSC-MVs were added to the MSC coculture system. One of the possibilities is that MSCs, as the feeder cells, secrete additional MVs in the coculture system. As we have demonstrated the beneficial effects of MSC-MVs on ex vivo expansion of CB, it is reasonable that exogenous addition of MSC-MVs to the coculture system resulted in the highest rate of CB expansion, compared with the other groups. In addition, several studies have demonstrated that MVs may in turn affect the behaviors of their parent cells [39, 40]. Thus it is also possible that MSC-MVs may promote the hematopoiesis-supporting effects of MSCs, and future investigations of the effects of MSC-MVs on their parent cells are worthwhile. Although the existing CB-MSC coculture system has been proved to be effective compared with a liquid culture system supplemented with a combination of cytokines [8], the long-term marrow-repopulating potential of HSCs may decline during ex vivo expansion with MSCs as a feeder layer [41]. Our results suggest that MSC-MVs may become a powerful additive to optimize the existing CB-MSC coculture expansion system.

The Wnt/*β*-catenin signaling pathway has been demonstrated to be essential for the regulation of HSPC functions [36]. Canonical Wnt signaling increases the expression of *β*-catenin in HSCs, promoting their self-renewal and inhibiting differentiation [42]. We investigated the effect of MSC-MVs on the expression of *β*-catenin in ex vivo-expanded CD34^+^ cells. The results showed that MSC-MVs increased the expression of *β*-catenin in expanded CD34^+^ cells compared with that in the control group. Although controversial, the loss of *β*-catenin has been demonstrated to result in an impaired ability of HSCs to self-renew [43]. It is possible that MSC-MVs exert their hematopoiesis-supporting effects through the Wnt/*β*-catenin pathway; however, this requires further investigation. Surprisingly, the expression of *β*-catenin in the MSC group decreased compared with that in the control group. It has been clearly demonstrated that the cargo of MVs is not randomly packaged in them [44]. In fact, there are precise mechanisms that are still largely unknown that regulate the sorting of bioactive cargo into MVs [45]. We speculate that MSC-MVs may be selectively enriched in proteins, mRNAs, or miRNAs that are involved in the Wnt/*β*-catenin pathway compared with their parent cells.

MVs have been demonstrated to be able to induce transcriptional and epigenetic reprogramming of their target cells by the horizontal transfer of their genetic packages (e.g., proteins, mRNAs, and miRNAs) [14, 15]. It is possible that ex vivo-expanded CD34^+^ cells can also be reprogrammed by MSC-MVs to generate greater numbers of primitive cells. The genetic packages of MVs are vital for the induction of reprogramming of their target cells. Numerous studies have demonstrated that the effects of MVs are at least partially abrogated after being treated with RNase or being heat-inactivated [14, 18, 46]. We believe that the bioactive cargo enclosed in MSC-MVs plays a key role in their hematopoiesis-supporting effects. miRNAs have been demonstrated to play pivotal roles in the regulation of hematopoiesis [22]. In the current study, we analyzed the miRNA expression profile of MSC-MVs and found that they contained miRNAs that are involved in the regulation of some characteristic functional properties of HSPCs. We also found 17 relatively highly expressed miRNAs in MSC-MVs that were predicted to target genes that have been reported to inhibit the Wnt/*β*-catenin pathway and are expressed in CB-derived HSCs. These results may explain why MSC-MVs could increase *β*-catenin expression in the expanded CD34^+^ cells. However, further studies are needed to verify this hypothesis. A number of other studies have also investigated the bioactive cargo of MSC-MVs and suggested that MSC-MVs contain proteins, mRNAs, and miRNAs that are involved in the regulation of stem cell-related functions [18, 47, 48]. These studies combined with our results may provide insight into the molecular mechanisms underlying the hematopoiesis-supporting effects of MSC-MVs.

MSC-MVs are considered to hold great therapeutic potential [17, 49]. They are naturally occurring vesicles that have been proved to have low immunogenicity [50]. Growing evidence suggests that MVs derived from human MSCs produce little toxicity or side effects in immunocompetent animals [13, 19, 20]. Several clinical trials using MVs derived from autologous dendritic cells to treat advanced cancer have indicated that MV therapy is safe and feasible [51]. In addition, attention has recently focused on engineering native MVs for therapeutic purposes [52]. It is possible to obtain engineered MVs enriched with therapeutic cargo that can target specific organs or tissues [53]. However, there are many issues that need to be addressed before the clinical application of MSC-MVs for the ex vivo expansion of CB. For example, in the current study, we only performed in vitro CFC and CAFC assays to evaluate the activities of HSPCs. It should be borne in mind that the true stem cell function can only be proved by in vivo transplantation assays where stem cells are capable of repopulating all blood lineages of irradiated recipients [34]. Therefore, it is necessary to evaluate whether MSC-MVs could improve the in vivo marrow-repopulating potential of the ex vivo-expanded cells. Additionally, the exact mechanism underlying the hematopoiesis-supporting effect of MSC-MVs remains unclear. Future studies should focus on the changes in target cells induced by MSC-MVs and the whole package of bioactive cargo enclosed in MSC-MVs. Finally, at present, differential centrifugation is the gold standard method to isolate MVs [38]. However, this process of purifying MVs is labor-intensive and time-consuming, so methods for the large-scale production of MSC-MVs need to be developed.

In conclusion, we demonstrated that MSC-MVs could improve the ex vivo expansion of CB-MNCs and CD34^+^ cells and generate greater numbers of primitive cells during this expansion. Our results provide insight into the potential use of MSC-MVs in the ex vivo expansion of CB.

## Supplementary Material

Supplementary materials include miRNAs expressed in MSC-MVs (Table S1) and the results of in vitro screen for the expansion of CB-MNCs with different concentrations of MSC-MVs (1, 5, 10, and 100 μg/ml) (Figure S1).

## Figures and Tables

**Table 1:** miRNAs expressed in MSC-MVs that are involved in regulation of hematopoiesis.

miRNA	Function	Targets	Ref.
miR-125a	Maintenance of self-renewal and differentiation balance in HSPCs	Unknown	[22]
miR-29a	Regulation of early hematopoiesis and myeloid commitment	HBP1	[23]
miR-223	Regulation of progenitor cell proliferation and granulocyte function	Mef2c	[24]
miR-21	Regulation of myelopoiesis	SMAD7	[25]
miR-451	Regulation of erythropoiesis	GATA-2	[26]
miR-144	Regulation of erythropoiesis	Klfd	[27]
miR-150	Modulating early B lymphocyte differentiation	c-Myb	[28]
miR-126	Regulation of primitive erythropoiesis	Vcam-1	[29]
miR-196b	Regulation of myelopoiesis	HOXA9, MEIS1	[30]
miR-125b	Regulation of stem cell pool size	Unknown	[22]
miR-181	Modulating T lymphocyte differentiation	DUSP6	[31]
miR-424	Regulation of monocytopoiesis	NFI-A	[32]
