# Supplementary material for: Mesenchymal Stem Cell-Derived Microvesicles Support Ex Vivo Expansion of Cord Blood-Derived CD34+ Cells
Source: Stem Cells Int. 2016 Mar 6;2016:6493241. doi: 10.1155/2016/6493241 (PMC4799819; doi:10.1155/2016/6493241)
Supplement: Supplementary file 1 — Supplementary materials include miRNAs expressed in MSC-MVs (Table S1) and the results of in vitro screen for the expansion of CB-MNCs with different concentrations of MSC-MVs (1, 5, 10, and 100 μg/ml) (Figure S1). [file 6493241.f1.pdf]

Supporting Information

Mesenchymal stem cell-derived microvesicles support ex vivo expansion of cord blood-derived $CD34^+$ cells

Hui Xie[1*], Li Sun[2*], Liming Zhang[3], Teng Liu[4], Li Chen[5], Aiqi Zhao[1], Qian Lei[1], Fei Gao[1], Qiubai Li[1], Anyuan Guo[4], Zhichao Chen[1], Hongxiang Wang[5]

[1]*Institute of Hematology, Union Hospital, Tongji Medical College, Huazhong University of Science and Technology, Wuhan 430022, P. R. China*

[2]*Department of Hematology, the Second Hospital of Hebei Medical University, Shijiazhuang 050000, P. R. China*

[3]*Department of Hematology, the Central Hospital of Jingzhou, Jingzhou 434020, P. R. China*

[4]*Department of Biomedical Engineering, College of Life Science and Technology, Huazhong University of Science and Technology, Wuhan 430074, P. R. China*

[5]*Department of Hematology, the Central Hospital of Wuhan, Wuhan 430012, P. R. China*

Correspondence should be addressed to: Zhichao Chen, chenzhichao@hust.edu.cn; and Hongxiang Wang, whitely1972@sina.com.

[*]These authors contributed equally to this work.

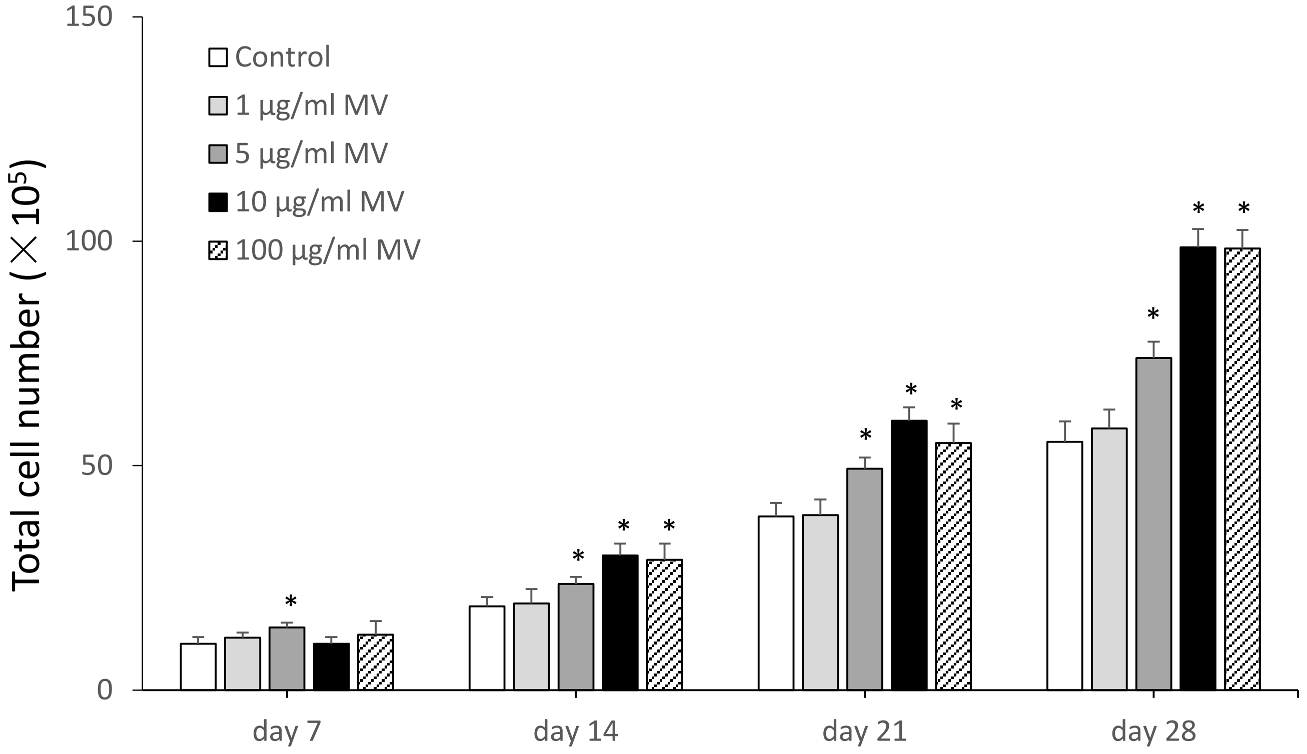

Figure S1: Ex vivo expansion of CB-MNCs with different concentrations of MSC-MVs. CB-MNCs were maintained under five different culture conditions: control, 1μg/mL MV, 5 μg/mL MV, 10 μg/mL MV and 100 μg/mL MV. Growth profile of TNCs (n = 3), *$p < 0.05$ compared with the control.

Table S1: miRNAs expressed in MSC-MVs. The expression levels of miRNAs were calculated as the base 2 logarithm of the nominalized signals.

miRNAs	expression level
hsa-miR-4281	14.567585
hsa-miR-1207-5p	13.745852
hsa-miR-1225-5p	13.164646
hsa-miR-1202	12.991837
hsa-miR-2861	12.682714
hsa-miR-638	12.50079
hsa-miR-320c	12.329145
hsa-miR-21	12.285721
hsv2-miR-H10	12.128811
hsa-miR-1275	12.089333
hsa-miR-4270	12.060837
hsa-miR-762	12.006063
hsa-miR-3656	11.941285
hsa-miR-1915	11.851605
hsa-miR-3665	11.754297
hsa-miR-630	11.669834
hsa-miR-150*	11.546194
hsa-miR-3162	11.470816
hsa-miR-3195	11.347115
kshv-miR-K12-3	11.337478
hsa-miR-4327	11.269712
hsa-miR-494	11.169283
hsa-miR-451	11.166111
hsa-miR-3663-3p	11.043634
hsa-miR-223	11.036649
hsa-miR-1268	11.017969
hsa-miR-3679-5p	10.8913555
hsv2-miR-H6	10.864179
hsa-miR-134	10.848331
hsa-miR-642b	10.780482
hsa-miR-1246	10.751949
hsa-miR-188-5p	10.640162
hsv1-miR-H17	10.603412
hsa-miR-720	10.57036
hsa-miR-575	10.416153
hsa-miR-3196	10.38527
hsa-miR-939	10.258823
hsa-miR-663	10.169552
hsa-miR-718	10.1124935

hsa-miR-4286	10.078846
hcmv-miR-UL70-3p	10.053999
hsv2-miR-H25	10.006031
hsa-miR-23a	9.842035
hsa-miR-940	9.708692
hsa-miR-483-5p	9.678314
hsa-miR-1471	9.655757
hsa-miR-4299	9.629159
hsa-miR-22	9.550125
hsa-miR-1274b	9.482363
hsa-miR-3141	9.457193
hsa-miR-572	9.376527
hsa-let-7a	9.324976
hsv2-miR-H24	9.271818
hsv1-miR-H6-5p	9.258377
hsa-miR-3198	9.251155
hsa-miR-29a	9.216732
hsa-miR-1224-5p	9.164385
hsv2-miR-H22	9.136539
hsa-miR-16	9.10329
hsa-miR-4298	9.059471
kshv-miR-K12-7*	9.049791
ebv-miR-BART13	9.004726
hsa-let-7f	8.997303
hsa-miR-135a*	8.951677
hsa-miR-1914*	8.949656
hsa-let-7b	8.887102
hsa-miR-27a	8.864937
hsa-miR-4271	8.844454
hsa-miR-513a-5p	8.801216
hsa-miR-3138	8.75045
hsv1-miR-H18	8.707052
hsa-miR-3188	8.669894
hsa-miR-125b	8.647974
hsa-miR-3667-5p	8.6212635
hbv-miR-B2RC	8.609455
hsa-miR-1305	8.591639
hsa-miR-3652	8.543315
hsv1-miR-H7*	8.509319
hsa-miR-24	8.476858
hsa-miR-1229	8.470226
hsa-miR-671-5p	8.44429
hsa-miR-1290	8.410376

hsa-miR-146a	8.379499
hsa-miR-142-3p	8.331952
hsa-let-7i	8.313329
hsa-miR-3648	8.303959
hsa-miR-100	8.270742
hsa-miR-1181	8.131459
hsa-miR-34a	8.099792
hsa-miR-125a-3p	8.075232
hsa-miR-601	8.04512
hsa-miR-3911	8.018745
hsa-miR-3937	7.984123
hsa-miR-874	7.9371705
hsa-miR-342-3p	7.926705
hsa-let-7g	7.915552
hsa-miR-765	7.887698
hsa-miR-345	7.865498
hsa-miR-150	7.860368
hsa-miR-2276	7.8568196
hsa-miR-1260b	7.8215027
hsa-miR-3131	7.8041406
hsa-miR-29b	7.7976923
hsa-miR-3156	7.771739
hsa-miR-15b	7.7524476
hsa-miR-1226*	7.735523
hsa-miR-3610	7.7213335
hsa-miR-3945	7.68521
ebv-miR-BART19-3p	7.649603
hsa-miR-320a	7.625454
hsa-miR-4284	7.59321
hsa-miR-320b	7.587061
hsa-miR-1280	7.5694084
hsa-miR-139-3p	7.553729
hsa-miR-3137	7.52622
hsa-miR-513b	7.509719
hsa-miR-574-5p	7.4982557
hsa-miR-3127	7.492097
hsa-miR-3125	7.4617014
hsa-miR-198	7.4252114
hsa-miR-548q	7.3945894
hsa-miR-557	7.348301
hsa-miR-320d	7.330092
hsa-miR-370	7.320868

hsa-miR-4306	7.26297
hsa-miR-622	7.2514753
hsa-miR-1183	7.2419906
hsa-miR-15a	7.210106
hsa-miR-199a-3p	7.1898584
hsa-miR-3917	7.1727705
hsa-miR-1288	7.158868
hsa-miR-19b	7.1449785
hsa-miR-320e	7.1105127
hsa-miR-3692*	7.1010222
hsa-miR-3194	7.0708694
hsa-miR-371-5p	7.024727
hsa-let-7d	6.9686575
hsa-miR-324-3p	6.96316
hiv1-miR-H1	6.940594
hcmv-miR-US4	6.9288077
hsa-miR-1182	6.9163275
hsa-miR-4257	6.9024043
hsa-miR-103	6.8786325
hsa-miR-29c	6.877446
hsa-miR-520b	6.8434925
hsv1-miR-H8	6.825449
kshv-miR-K12-5*	6.792757
hsa-miR-27b	6.7865763
hsa-miR-193b*	6.7807984
hsa-miR-25	6.7573423
hsa-miR-26b	6.727973
hsa-miR-221	6.725342
hsa-miR-149*	6.701896
hsa-miR-107	6.682109
hsa-miR-202	6.650899
hsa-miR-877	6.6452036
hsa-miR-1249	6.640189
hsa-miR-20a	6.6283836
hsa-miR-564	6.5982594
hsa-miR-4322	6.5616736
hsa-miR-513c	6.5305376
hsa-miR-3682	6.525962
hsa-miR-1973	6.5121965
hsa-let-7c	6.5089855
hsa-miR-1228	6.4941483
hsa-miR-181a	6.4804735
hsa-miR-887	6.464864

hsa-miR-1274a	6.445038
hsa-miR-623	6.4438314
hsa-miR-3621	6.4202967
hsa-miR-1260	6.3744287
hsa-miR-629*	6.3666544
hsa-miR-23b	6.3469987
hsa-miR-4314	6.2705975
hsv1-miR-H16	6.259321
hsa-miR-26a	6.246234
hsa-let-7e	6.216729
ebv-miR-BART7	6.206436
hsa-miR-92a	6.192953
hsa-miR-3907	6.183742
hsv1-miR-H1	6.171818
hsa-miR-498	6.164486
hsa-miR-365	6.146815
hsa-miR-892b	6.1217833
hsa-miR-769-3p	6.1114383
hsa-miR-4253	6.088073
hsa-miR-3132	6.076616
hsa-miR-155	6.0633006
hsa-miR-4304	6.0333924
hsa-miR-516a-5p	6.0256453
hsa-miR-520e	6.021188
hsa-miR-423-5p	5.980315
hsa-miR-936	5.9676275
hsa-miR-3190	5.961391
hsa-miR-106b	5.9278445
hsa-miR-145	5.918894
hsa-miR-486-5p	5.914959
hsa-miR-30b	5.9035883
hsa-miR-500a	5.890527
hsa-miR-514b-5p	5.889735
hsa-miR-659	5.8651314
hsa-miR-3646	5.8547306
hsa-miR-1225-3p	5.8484735
hsa-miR-30e	5.8215866
hsa-miR-30c-1*	5.8180933
hsa-miR-3622a-5p	5.7951317
hsa-miR-199a-5p	5.7924604
hsa-miR-584	5.783854
hsa-miR-30d	5.776058
hsa-miR-19a	5.7480183

hsa-miR-3202	5.7275953
hsa-miR-1299	5.697763
hsa-miR-378	5.6829734
hbv-miR-B20	5.6766653
hsa-miR-760	5.656721
hsa-miR-3934	5.650096
hsa-miR-425	5.648071
hsa-miR-17	5.6313367
hsv2-miR-H9-5p	5.6244364
hsa-miR-130a	5.6228228
hsv1-miR-H14-3p	5.6060157
hsa-miR-144	5.588946
hsa-miR-1273e	5.5699577
hsa-miR-1972	5.56094
kshv-miR-K12-8*	5.5450654
hsa-miR-3713	5.5366144
hsv2-miR-H2	5.524804
hsa-miR-378b	5.5095763
hsa-miR-126	5.492161
hsa-miR-3620	5.470476
hsa-miR-4294	5.4573026
hbv-miR-B4	5.4477577
hsa-miR-10b*	5.4315786
hsa-miR-1234	5.4243937
hsa-miR-193a-5p	5.406678
hsa-miR-424	5.398101
hsa-miR-331-3p	5.3856583
hsv1-miR-H15	5.383128
bkv-miR-B1-5p	5.36899
hsa-miR-30a	5.367517
hsa-miR-187*	5.333323
hsa-miR-3154	5.30785
hsa-miR-142-5p	5.2978783
hsa-miR-1469	5.2968225
kshv-miR-K12-10b	5.2911406
hsa-miR-3161	5.263558
hsa-miR-93	5.257251
hsa-miR-1306	5.2527676
hsa-miR-662	5.2386284
hsa-miR-490-5p	5.2133293
hsa-miR-501-5p	5.206587
hsa-miR-374a	5.159381
hsa-miR-422a	5.15327

hsa-miR-185	5.139147
hsa-miR-4282	5.1300755
hsa-miR-23a*	5.1223097
hsa-miR-148a	5.1093135
hsa-miR-1208	5.095356
hsa-miR-214	5.0789213
hsa-miR-3187	5.066514
hsa-miR-151-5p	5.061845
hsa-miR-125b-2*	5.0501995
hsa-miR-1323	5.02599
hsa-miR-210	4.99925
hsa-miR-1295	4.990408
hsa-miR-3622b-5p	4.986885
hsa-miR-583	4.9794745
hsa-miR-3659	4.975812
hsa-miR-298	4.9730263
hsa-miR-711	4.9592266
hsa-miR-610	4.9569855
hsa-miR-30c	4.9361496
hsa-miR-181b	4.931223
hsa-miR-1238	4.924039
hsa-miR-1273c	4.9215536
hsa-miR-140-3p	4.8835716
hsa-miR-3654	4.8668785
hsa-miR-3926	4.85363
hsa-miR-374b	4.8428397
hsa-miR-3925	4.840644
hsa-miR-4259	4.8332644
hsa-miR-605	4.8253736
hsa-miR-193b	4.821718
hsa-miR-3147	4.797043
hsa-miR-373*	4.7802134
hsa-miR-3150b	4.776926
hsa-miR-512-3p	4.7693586
hsa-miR-125a-5p	4.7462516
hsa-miR-3605-5p	4.73293
hsa-miR-361-5p	4.723005
hsa-miR-617	4.7151737
hsa-miR-212	4.7085743
hsa-miR-30c-2*	4.706713
hsa-miR-197	4.68123
hsa-miR-99b*	4.669672
hsa-miR-376c	4.652832

hsa-miR-191*	4.62821
hsa-miR-146b-5p	4.6192274
hsa-miR-3180-3p	4.605529
hsa-miR-3666	4.5949802
hsa-miR-140-5p	4.5826044
hsv2-miR-H3	4.5705557
hsa-miR-3689a-5p	4.5536947
hsv1-miR-H3*	4.53466
hsa-miR-3617	4.5257034
hsa-miR-3200-5p	4.5151615
hsv1-miR-H2*	4.497773
hsa-miR-296-5p	4.482547
hsa-miR-99b	4.478032
hsa-miR-101	4.4575768
hsa-miR-130b	4.4354644
hsa-miR-4313	4.410917
hsa-miR-3660	4.3924875
hsa-miR-4269	4.384628
hsa-miR-1254	4.3772244
hsa-miR-3189	4.360774
hsa-miR-339-3p	4.3579097
hcmv-miR-US33-5p	4.352947
hsa-miR-99a	4.335063
hsa-miR-602	4.320478
hsa-miR-636	4.302271
hsa-miR-1287	4.2796936
hsa-miR-3185	4.259828
hsa-miR-3182	4.246178
hsa-miR-665	4.2421007
hsa-miR-138-2*	4.2405
hsa-miR-1281	4.225437
hsa-miR-766	4.2144012
hsa-miR-708	4.2044234
hsa-miR-3680*	4.1917934
hsa-miR-132	4.186405
hsa-miR-3676	4.1730585
hsa-miR-148b	4.163746
hsa-miR-4251	4.156004
hsa-miR-20b	4.1442237
hsa-miR-196a	4.1293497
hsa-miR-425*	4.1112337
hsa-miR-590-5p	4.0975156
hsv1-miR-H3	4.092599

hsa-miR-550a	4.08558
hsa-miR-3923	4.056779
hsa-miR-193a-3p	4.0350733
hsa-miR-513a-3p	4.001229
hsa-miR-614	3.9935117
hsa-miR-1307	3.9724243
hsa-miR-877*	3.9487581
hsa-miR-770-5p	3.9405165
hsa-miR-660	3.9336748
hsa-miR-1237	3.9151406
hsa-miR-98	3.894874
hsa-miR-28-3p	3.8910751
hsa-miR-1825	3.879572
hsa-miR-196b	3.8660698
hsa-miR-663b	3.8615017
hsa-miR-302c*	3.8569658
ebv-miR-BHRF1-1	3.844483
hsa-miR-33b*	3.8391492
hsa-miR-3177	3.8196063
hsa-miR-222	3.81109
hsa-miR-1303	3.7894883
hsa-miR-3938	3.7735357
hsa-miR-1273d	3.764999
hsa-miR-526b	3.7525933
hsa-miR-138	3.748384
hsa-miR-377	3.7403884
hsa-miR-376a	3.7331734
hsa-miR-10b	3.7145371
hsa-miR-184	3.710299
hsa-miR-4260	3.7029305
hsa-miR-3197	3.673366
hsa-miR-3663-5p	3.6415882
hsa-miR-596	3.632085
hsa-miR-143	3.629878
hsa-miR-542-5p	3.6185164
hsa-miR-654-5p	3.6120281
hsa-miR-509-3-5p	3.6052294
hsa-miR-1180	3.5953264
hsa-miR-151-3p	3.5686638
hsa-miR-128	3.5363293
hsa-miR-34b*	3.525735
hsa-miR-631	3.5022857
hsa-miR-186	3.4942274

hsa-miR-378c	3.4898176
hsa-miR-365*	3.4818244
hsa-miR-205	3.4575315
kshv-miR-K12-8	3.4413679
hsa-miR-1	3.421696
hsa-miR-4261	3.4072268
hsa-miR-340	3.403561
hsa-miR-4311	3.3917136
hsa-miR-3170	3.3854876
hsa-miR-1911	3.358883
hsa-miR-574-3p	3.3354795
hsa-miR-3651	3.3352032
hsa-miR-127-3p	3.3319952
hsa-miR-30b*	3.315051
hsa-miR-1285	3.2940593
hsa-miR-338-3p	3.2902296
hsa-miR-338-5p	3.2789385
hsa-miR-664*	3.2303243
hsa-miR-152	3.2204356
hsa-miR-1261	3.1965456
hsa-miR-1321	3.1883433
hsa-miR-652	3.1404533
hsa-miR-4323	3.1318564
hsa-miR-3178	3.1241574
hsa-miR-324-5p	3.118023
hsa-miR-199b-5p	3.1063182
hsa-miR-661	3.0946808
hsa-miR-21*	3.0855656
hsa-miR-10a	3.0808969
hsa-miR-505	3.0599911
hsa-miR-28-5p	3.049326
hsa-miR-3653	3.044369
hsa-miR-566	3.0398333
hsa-miR-516b	3.0262516
hsa-miR-133b	2.9991283
hsa-miR-3163	2.992115
hsa-miR-492	2.9880893
hsa-miR-424*	2.9743433
hsa-miR-933	2.9558759
hsa-miR-2277-3p	2.9057229
hsa-miR-4317	2.8924837
hsa-miR-4324	2.8793259
hsa-miR-532-5p	2.8593621

hsa-miR-301a	2.8487623
hsa-miR-186*	2.837029
hsa-miR-192	2.8177302
hsa-miR-129-5p	2.8019385
hsa-miR-1915*	2.7918816
hsa-miR-409-3p	2.7807622
hsa-miR-648	2.767327
hsa-miR-361-3p	2.7650313
hsa-miR-3174	2.746513
hsa-miR-658	2.7367568
hsa-miR-487b	2.7329187
hsa-miR-371-3p	2.7091498
hsa-miR-342-5p	2.6909904
hsa-miR-508-5p	2.6792846
hsa-miR-3124	2.6667027
hsa-miR-628-5p	2.6522994
hsa-miR-195	2.6393867
hsa-miR-500a*	2.6313558
hsa-miR-3679-3p	2.6140695
hsa-miR-519e*	2.5904584
hsa-miR-432	2.548689
hsa-miR-509-5p	2.5264719
hsa-miR-3915	2.4814987
hsa-miR-3181	2.4773948
hsa-miR-362-3p	2.4514635
jcv-miR-J1-5p	2.4285305
hsa-miR-1243	2.3974748
hsa-miR-3180-5p	2.3835335
hsa-miR-136	2.36044
hsa-miR-551b	2.3465629
hsa-miR-495	2.3232994
hsa-miR-501-3p	2.1862235
hsa-miR-34c-3p	2.154354
hsa-miR-650	2.1348248
hsa-miR-934	2.1268258
hcmv-miR-UL36	2.1132197
hsa-miR-632	2.0501277
hsa-miR-200a*	2.0045772
ebv-miR-BART16	1.8961444
hcmv-miR-US25-1*	1.889236
hsa-miR-639	1.8706405
hsa-miR-551b*	1.8673565
hsa-miR-194*	1.7747277

hsa-miR-125b-1*	1.7510183
